# Fragments*Read by Flora A. Waddell, M. D., of Wauseon, Ohio, at the meeting of the International Hahnemannian Association, Niagara Falls, N. Y., June, 1888.

**Published:** 1888-08

**Authors:** Flora A. Waddell


### FRAGMENTS.*

MR. PRESIDENT, LADIES, AND GENTLEMEN:—The subject under consideration at present is the diseases of women and children. They are too many to enumerate here. Some are avoidable by proper living and care; others are dependent on the same chronic miasm that curses so many of the human race. I treat my cases of this class, just as I do all other diseases, by the remedy that covers the totality of the symptoms. I occasionally meet with cases that are devoid of symptoms. One of the professors in lecturing on diseases of women said, When you have a case of this kind give them Sepia and wait. I do not know how this would work, as I have never tried it. Will give two what I call symptomless cases, and their treatment. Case first, young lady, twenty-three years of age, has good health in every way, except profuse menses, which commenced at the age of sixteen to be very bad. Would last eight to ten days and leave her very weak; would not recover strength from one period to the next; did not suffer any pain; could not give a symptom or

---

* Read by Flora A. Waddell, M. D., of Wauseon, Ohio, at the meeting of the International Hahnemannian Association, Niagara Falls, N. Y., June, 1888.

condition, except what I have mentioned. I did not know what to do with it. There was nothing characteristic or peculiar about it; was not used to prescribing for a *name*. After thinking the matter over awhile, came to the conclusion to try Sabina. Its power over menorrhagia is well known to all. Gave a dose of the 200x every night for a week before menstruation. Placebo. Passed the period with five days' flow not so profuse; next month repeated the same. Reported four days' flow and not bad. Had gained in flesh and strength. Next month gave Sac. Lac.; reported all right, result all that could be asked for. Considers herself well at the present time. Case second, married lady. Had menorrhagia from first menstruation, never been pregnant. Came to be treated for sterility. No marked symptoms. Gave Sabina$^{200x}$ same as the other case. In three months menstruation seemed to be normal. One year later was delivered of a fine boy. This lady was a Jewess, and according to their religion sterility is considered a curse. The curse was lifted and all were happy. Case third *had* a symptom so peculiar that I have recorded it. I had never seen it before. I only mention it on account of its peculiarity. Lady, fifty-three years of age. Cancer of left breast. Case was in the last stage when I was called; only lived a few weeks after I took charge of her. Whenever she would try to drink, would have to take very small sips at a time for want of breath. Would seem to want a quantity of liquid at a time, but would get so tired would have to desist. Under Nitrum, in Hering, found the condition described very accurately. Gave a few doses of the 30x, the only potency I had of it, which relieved her entirely at the time and only one slight return of it afterward. This may be of use to some one as a verified symptom of Nitrum, a remedy which we seldom have use for. Case fourth, ovary of left side affected. Pain always in the heart when the ovary ached. Pains sharp and cutting, come on about a week before menses, growing worse until flow began, then easier until next month, when they would return again. This had been of over two years' duration. Naja is the only remedy having this symptom that I know of. Gave it in the twelfth, dose four times a day. Entire relief followed. I have verified this symptom of Naja several times. As to diseases of children, I have very little to say that would be of benefit to any one here.

One case or two might be mentioned. Child had been ailing over four months when brought to me. Parents lived some thirty miles away. Family history good, so far as I could learn. Child had never been sick until this attack. Could not get a very satisfactory account of its first sickness; was now thirteen months old, and had been treated allopathically by three different doctors. Their verdict was death. It could not hold its head up; eyes turned back in its head; could see but very little by holding an article above its eyes. Would cry out every few minutes and put its hand to one side of its head, almost the only motion it could make. Head very large, exuding a sour sweat; bowels constipated, appetite abnormal; could not eat enough to satisfy it. Teeth very slow coming, and one side of the body more dormant than the other. Constantly boring its head backward into the pillow.

Gave a dose of Nux vomica$^{200x}$ and watched the child awhile each day for three days; came to the conclusion that Apis covered more of the symptoms than any other remedy. Gave a dose of 1m dry on the tongue, followed by unmedicated pills four times a day.

In three days improvement seemed fairly begun. Parents reported once in two weeks. The improvement was steady and sure. Only one dose more of Apis was given and that was CMM. In a little over three months there was scarcely a trace of the disease left. Have not heard since.

Case second. Child two years old. Would cry all night, about daylight would go to sleep and sleep all the forenoon. Had kept this up for over two weeks. Parents could not find any cause for it. The rest of the day she would eat and play all right. Examined her thoroughly; could find nothing wrong; asked if she was in pain anywhere; she said no. Gave a dose of Syphilinum, as it has this symptom of time of aggravation so marked. It gave no relief. In looking up another case I came to the symptom under Calcarea carb. Gave a dose of 200x every night for three nights. After the first dose had no more trouble; slept all night. That was six months ago. Last week they asked for medicine again. The same conditions returning. Gave one dose of MM, which relieved again. Have not found any cause for the trouble yet. If any one here has had a similar experience should like to hear from them.

